# Emergency admission parameters for predicting in-hospital mortality in patients with acute exacerbations of chronic obstructive pulmonary disease with hypercapnic respiratory failure

**DOI:** 10.1186/s12890-021-01624-1

**Published:** 2021-08-06

**Authors:** Lan Chen, Lijun Chen, Han Zheng, Sunying Wu, Saibin Wang

**Affiliations:** 1 Nursing Education Department, Affiliated Jinhua Hospital, Zhejiang University School of Medicine, Jinhua Municipal Central Hospital, Jinhua, Zhejiang Province China; 2 Emergency Department, Affiliated Jinhua Hospital, Zhejiang University School of Medicine, Jinhua Municipal Central Hospital, Jinhua, Zhejiang Province China; 3 Department of Respiratory Medicine, Affiliated Jinhua Hospital, Zhejiang University School of Medicine, Jinhua Municipal Central Hospital, Jinhua, Zhejiang Province China

**Keywords:** Acute exacerbation of chronic obstructive pulmonary disease, Hypercapnic respiratory failure, Nomogram, Mortality risk

## Abstract

**Background:**

Acute exacerbation of chronic obstructive pulmonary disease (AECOPD) is a common presentation in emergency departments (ED) that can be fatal. This study aimed to develop a mortality risk assessment model for patients presenting to the ED with AECOPD and hypercapnic respiratory failure.

**Methods:**

We analysed 601 participants who were presented to an ED of a tertiary hospital with AECOPD between 2018 and 2020. Patient demographics, vital signs, and altered mental status were assessed on admission; moreover, the initial laboratory findings and major comorbidities were assessed. We used least absolute shrinkage and selection operator (LASSO) regression to identify predictors for establishing a nomogram for in-hospital mortality. Predictive ability was assessed using the area under the receiver operating curve (AUC). A 500 bootstrap method was applied for internal validation; moreover, the model’s clinical utility was evaluated using decision curve analysis (DCA). Additionally, the nomogram was compared with other prognostic models, including CRB65, CURB65, BAP65, and NEWS.

**Results:**

Among the 601 patients, 19 (3.16%) died during hospitalization. LASSO regression analysis identified 7 variables, including respiratory rate, PCO_2_, lactic acid, blood urea nitrogen, haemoglobin, platelet distribution width, and platelet count. These 7 variables and the variable of concomitant pneumonia were used to establish a predictive model. The nomogram showed good calibration and discrimination for mortality (AUC 0.940; 95% CI 0.895–0.985), which was higher than that of previous models. The DCA showed that our nomogram had clinical utility.

**Conclusions:**

Our nomogram, which is based on clinical variables that can be easily obtained at presentation, showed favourable predictive accuracy for mortality in patients with AECOPD with hypercapnic respiratory failure.

**Supplementary Information:**

The online version contains supplementary material available at 10.1186/s12890-021-01624-1.

## Background

Chronic obstructive pulmonary disease (COPD) is a common chronic respiratory disease. It has a chronic and progressive course that is often punctuated by "exacerbations", which are clinically defined as exacerbations of respiratory symptoms. Specifically, they include dyspnoea, cough and sputum production, and increased sputum purulence [1]. Acute exacerbation of COPD (AECOPD) is a common presentation at emergency departments (ED) that is associated with a mortality rate of 1.8–8% [2–4]. The occurrence of hypercapnia in patients with COPD might be indicative of the presence of hypoventilation syndrome, which is associated with a negative prognosis [5, 6]. However, there are significant among-patient differences in the severity, evolution, and outcome of exacerbations. Specifically, some patients make a full recovery within a short time period, while others may die [7]. There is a need for an effective tool for helping emergency care providers promptly determine the mortality risk of patients with AECOPD to inform treatment decisions.

There have been numerous studies on the prognostic risk factors for AECOPD [6–13], including age, body mass index (BMI), lung function tests, blood gas analysis, inflammation-related indicators, and smoking history. However, there have been inconsistent reports regarding the predictors of poor outcomes in patients with AECOPD due to the heterogeneity across the previous studies with respect to the assessed variables, study population, and outcomes of interest [7]. Moreover, emergency patients, especially those in critical care, lack immediately available data regarding lung function, BMI, and the 6-min walk test. Additionally, individual risk predictors cannot allow systematic risk assessment. Scoring systems for assessing the criticality of patients with AECOPD include the medical research council (MRC) scale [11] and Acute Physiology and Chronic Health Evaluation-II score (APACHE-II) [14]. The MRC dyspnoea scale is a questionnaire for assessing long-term mortality that is comprised of five statements regarding perceived breathlessness. The APACHE-II assesses numerous parameters collected within 24 post-admission hours. However, it is mainly suitable for the intensive care unit (ICU) rather than ED. CURB65, CRB65, BAP65, and NEWS, which mainly involve mental status, respiratory rate, oxygen saturations, pulse, blood pressure, age, BUN level, etc., can be used to predict AECOPD-associated mortality in ED given the simple structure and data availability [2–4, 15]. However, these scoring systems have been rarely studied; moreover, they have a poor or modest value for predicting mortality (AUC 0.606–0.744) [2–4, 15–17]. Additionally, it is difficult for these systems to yield an individual survival assessment.

This study aimed to extensively collect predictors readily available from patients with AECOPD presented at the ED, and subsequently develop and validate a nomogram for individual mortality risk prediction in patients with AECOPD with hypercapnic respiratory failure.

## Materials and methods

### Patients

This retrospective cohort study was approved by the Ethics Committee of Jinhua Municipal Central Hospital (No. 2020-254). Since the data were anonymous, the requirement for informed consent was waived. The study was conducted in accordance with the principles of the Declaration of Helsinki. We enrolled 601 consecutive patients with AECOPD with hypercapnic respiratory failure who underwent treatment at an ED between January 2018 and September 2020. The inclusion criteria were as follows: (1) patients with a primary diagnosis of AECOPD or “respiratory failure” and a secondary diagnosis of AECOPD and (2) PCO_2_ arterial ≥ 50 mmHg. AECOPD was defined as (1) a COPD history, diagnosed according to the GOLD guideline [18] and (2) acute-onset sustained worsening in baseline dyspnoea, cough, and/or sputum that warranted a change in regular medication [18]. The exclusion criteria were as follows: (1) patients requiring immediate cardiopulmonary resuscitation; (2) patients who had been admitted to the neurology and cardiology wards for other acute diseases, including cerebral haemorrhage, myocardial infarction, poisoning, etc.

### Endpoints

The primary endpoint was hospital mortality. For sensitivity analysis, the secondary endpoint was the need for invasive mechanical ventilation in the ED or ICU admission.

### Collection of clinical data by reviewing medical records

We selected potential candidate variables, including patient demographics, data available in ED, and risk factors identified in previous studies. Specifically, we collected demographic factors (age and sex), initial vital signs (temperature, pulse, respiratory rate, systolic blood pressure, and diastolic blood pressure), consciousness (“alert, verbal, pain, unresponsive”) obtained at ED admission, initial laboratory findings obtained in ED, and underlying comorbid conditions. Regarding laboratory tests, potential predictive variables included results from blood gas analysis, haematology tests, routine chemical tests, coagulation function, troponin T, and N-terminal pro-brain natriuretic peptide levels. Considered comorbidities included pneumonia, underlying cardiopulmonary disease, hypertension, diabetes, chronic renal failure, chronic liver disease, history of malignant neoplasm, and other chronic conditions identified from the medical record review. CRB65, CURB65, BAP65, and NEWS were calculated based on the initial available data. At the study site, the initial vital signs were recorded in the emergency triage system, which comprises the electronic medical records. Further, data regarding laboratory tests and clinical interventions could be accessed from the electronic medical records.

### Statistical analysis

The patients’ baseline characteristics were summarized using descriptive statistics. Categorical and continuous variables were presented as number (percentages) and mean ± standard deviation or median (interquartile), respectively. Between-group comparisons of normally and non-normally distributed continuous variables were performed using Student’s t-test and the Mann–Whitney U test, respectively. Among-group comparisons of categorical variables were performed using Pearson’s chi-squared test or Fisher’s exact test, as appropriate.

Among the 80 variables, predictors were selected using the least absolute shrinkage and selection operator (LASSO) logistic regression algorithm, which is suitable for the regression of high-dimensional data [19]. Logistic regression analysis was used for establishing a model for predicting death during hospitalization and a nomogram was constructed. Based on clinical considerations, we also added the variable whether patient with pulmonary infection or not into the model construction. The area under the curve (AUC) was calculated to quantify the discrimination performance of the nomogram; further, internal validation was performed through bootstrapping with 500 iterations. Decision curve analysis (DCA) was performed to estimate the clinical utility of the nomogram by calculating the net benefits at different threshold probabilities. All statistical analyses were conducted using EmpowerStats (www.empowerstats.com), R 3.5.1 (www.r-project.org), and MedCalc version 15 for Windows (MedCalc Software bvba). Statistical significance was set at a two-sided *P* value < 0.05.

## Results

**Fig. 1:**
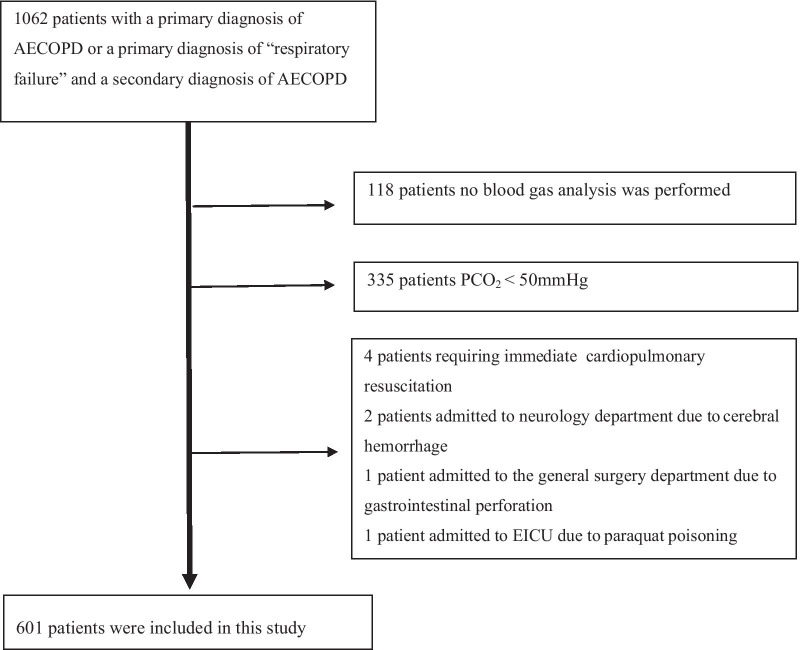
The study flow chart of the patient cohort

Figure 1 presents the study flowchart. Initially, 1062 patients were recruited; among them, 601 patients remained after applying the exclusion criteria. Table 1 and Additional file 1: Table S1 show the patient characteristics. Among the 601 patients, 213 (35.44%) received non-invasive mechanical ventilation, 137 (22.80%) received invasive mechanical ventilation, 6 (1.00%) died in ED, 172 (28.62%) were admitted to the ICU, and 19 (3.16%) died during hospitalization. Univariate analysis revealed significant differences in temperature, respiratory rate, level of consciousness, PH, PCO_2_, BE, lactic acid, lactic dehydrogenase, potassium, magnesium, albumin, albumin/globulin ratio, blood amylase, BUN, C-reactive protein, white blood cell count, neutrophils count, red blood cell count, haematocrit, haemoglobin, platelet distribution width (PDW), international normalized ratio, prothrombin time, and D-dimer levels between the death and survival. Furthermore, we collected the results of lung function tests. However, among the 601 patients, only eight lung function tests were performed within 6 months before admission. Therefore, these data were not included in the statistical analysis.

**Table 1:** Primary baseline demographic and clinical characteristics of the participants

Variable	Death during hospitalization		*P*
	Yes (n = 19)	No (n = 582)	
*Baseline characteristics*			
Age (years)	77.9 ± 11.6	76.2 ± 10.2	0.476
Male, n (%)	12 (63.2)	413 (71.0)	0.462
*Admissions for AECOPD in year prior, n (%)*			0.724
0	444 (76.3)	16 (84.2)	
1	90 (15.5)	2 (10.5)	
≥ 2	48 (8.2)	1 (5.3)	
*Monitoring parameters at admission*			
Temperature (degrees Celsius)	36.2 ± 0.9	36.9 ± 0.8	< 0.001
Respiratory rate (beat/min)	27.4 ± 6.3	22.7 ± 6.2	0.001
Level of consciousness using the AVPU system, n (%)			< 0.001
A	8 (42.1)	486 (83.6)	
V	3 (15.8)	35 (6.0)	
P	2 (10.5)	27 (4.6)	
U	6 (31.6)	33 (5.7)	
***Laboratory findings***			
PH	7.2 ± 0.1	7.3 ± 0.1	< 0.001
PCO_2_ (mmHg)	87.4 ± 38.2	71.5 ± 17.1	< 0.001
PO_2_ (mmHg)	64.4 (53.1–91.2)	60.4 (45.0–81.1)	0.182
PaO_2_/FiO_2_	176.4 (99.0–240.0)	191.1 (139.0–253.4)	0.179
BE (mmol/L)	5.2 (-5.2–9.4)	8.3 (-4.7–12.3)	0.010
Lactic acid (mmol/L)	2.7 (1.4–4.8)	1.4 (0.9–2.4)	< 0.001
Lactic dehydrogenase (IU/L)	335.1 (246.8–600.2)	265.0 (208.4–418.5)	0.002
Potassium (mmol/L)	4.6 ± 0.7	4.2 ± 0.7	0.005
Magnesium (mmol/L)	1.0 ± 0.1	0.9 ± 0.1	0.002
Albumin (g/L)	32.6 ± 5.2	36.0 ± 5.2	0.006
Albumin/Globulin ratio	0.9 ± 0.3	1.1 ± 0.3	0.012
Blood amylase (U/L)	86.0 (60.5–116.5)	63.0 (45.0–84.8)	0.027
Creatinine (μmol/L)	81.6 (66.5–161.9)	74.5 (58.4–97.9)	0.087
Blood urea nitrogen (mmol/L)	10.5 (7.9–14.2)	6.5 (4.9–8.7)	< 0.001
C-reactive protein (mg/L)	47.5 (23.9–81.6)	18.4 (4.7–56.0)	0.033
White blood cell count (× 10^9^/L)	14.7 ± 6.4	10.3 ± 6.3	0.003
Lymphocyte count (× 10^9^/L)	1.2 (0.7–1.8)	0.9 (0.6–1.3)	0.056
Neutrophils count (× 10^9^/L)	11.7 ± 4.9	8.3 ± 5.7	0.011
Red blood cell count (× 10^12^/L)	4.0 ± 0.5	4.7 ± 2.9	0.001
Haematocrit (%)	37.8 ± 3.9	42.7 ± 7.4	0.004
Haemoglobin (g/L)	116.4 ± 12.9	135.8 ± 23.8	< 0.001
Platelet count (× 10^9^/L), n (%)			0.108
< 100	2 (10.5)	53 (9.5)	
≥ 100, < 300	12 (63.2)	444 (79.6)	
≥ 300	5 (26.3)	61 (10.9)	
Mean platelet volume (fL)	10.1 ± 1.0	10.6 ± 1.2	0.073
Platelet distribution width (%)	11.2 ± 2.1	12.5 ± 2.9	0.043
*Comorbidities, n (%)*			
Pneumonia	7 (36.8)	267 (45.9)	0.437
Hydropneumothorax	0 (0.0)	4 (0.7)	1.000
Heart failure, coronary heart disease	3 (15.8)	137 (23.5)	0.432
Hypertension	0 (0.0)	20 (3.4)	0.411
Diabetes	0 (0.0)	4 (0.7)	0.717
Respiratory cancer	1 (5.3)	8 (1.4)	0.170
Chronic kidney failure	0 (0.0)	3 (0.5)	1.000
Pulmonary thromboembolism	2 (0.3)	0 (0.0)	0.798
Deep vein thrombosis	2 (0.3)	0 (0.0)	0.798
*Intervention measures, n (%)*			
Non-invasive mechanical ventilation	6 (31.6)	207 (35.6)	0.721
Invasive mechanical ventilation	11 (57.9)	126 (21.6)	< 0.001
*Outcomes, n (%)*			
Death in emergency department	6 (31.6)	0 (0.0)	< 0.001
ICU admission	11 (57.9)	161 (27.7)	0.004

**Fig. 2:**
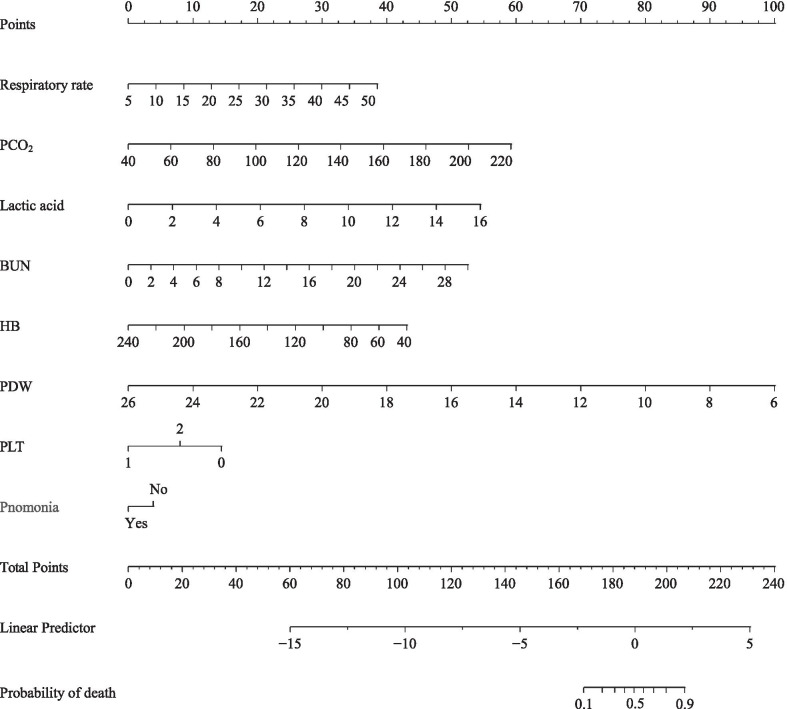
A nomogram for predicting death during hospitalization among patients who presented to the ED with AECOPD. First, find the point for each predictor of an individual on the uppermost rule. Second, determine the sum of all points and find the “total points” on the rule. Finally, the corresponding predicted probability of death during hospitalization can be found on the lowest rule. For example, a patient with: (1) respiratory rate of 30 breath/min (22 point), (2) lactic acid 10 mmol/L (33 point), (3) PCO_2_ 80 mmHg (13 point), 4) BUN 16 mmol/L (28 point), (5) haemoglobin 100 g/L (30 point), (6) platelet distribution width 16% (50 point), (7) platelet count 80 × 10^9^/L (15 point), and (8) with pneumonia (0 point) would have a total score of 191 points and a risk of death around 60%

**Fig. 3:**
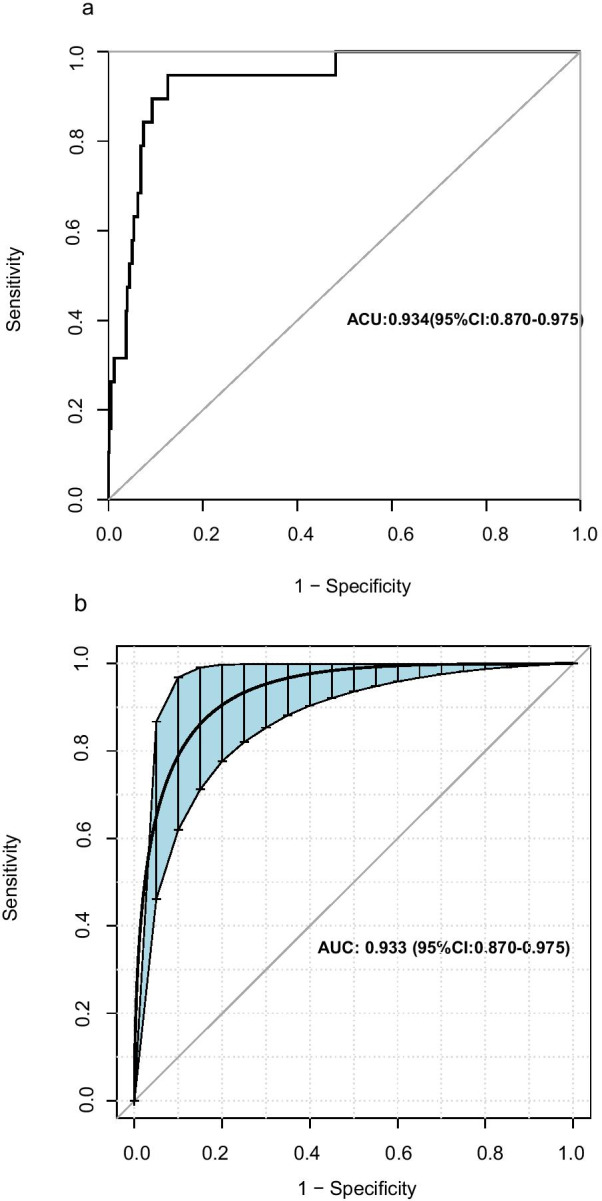
Receiver operating characteristic curves of the nomogram and internal validation. AUC **a** shows the discrimination of the model. AUC **b** of the internal validation. The blue shading denotes the bootstrap estimated 95% confidence interval with the AUC. The corresponding 95% confidence interval estimate is highlighted in black text

Among 80 variables, 7 non-zero coefficients were screened through LASSO logistic regression analysis using the minimum criteria, including respiratory rate, lactic acid, PCO_2_, BUN, haemoglobin, platelet count, and PDW. We constructed a nomogram incorporating these seven predictors and the the variable of concomitant pneumonia (Fig. 2). An AUC of 0.940 (95% CI 0.895–0.985) indicated good discrimination by the nomogram for predicting the mortality risk in the patients (Fig. 3a). The AUC for the internal validation model was 0.933 (95% CI 0.870–0.975) (Fig. 3b).

**Fig. 4:**
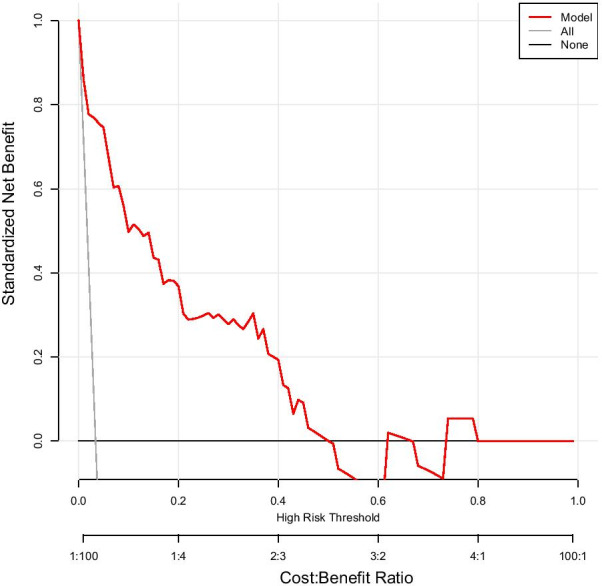
Decision curve analysis for the nomogram. The y-axis and x-axis represent the net benefit and threshold probability, respectively. The threshold probability is where the expected treatment benefit is equal to the expected benefit of avoiding treatment. The red solid line represents the nomogram. The decision curve indicates that for a threshold probability of 4–55%, applying this predictive model could add net benefits compared with treating either all or no patients

Figure 4 presents the DCA for the nomogram. DCA showed that when the model for assessing the in-hospital mortality risk ranged from 0.04 to 0.55, the nomogram added more net benefits than the “treat all” or “treat none” strategies.

**Fig. 5:**
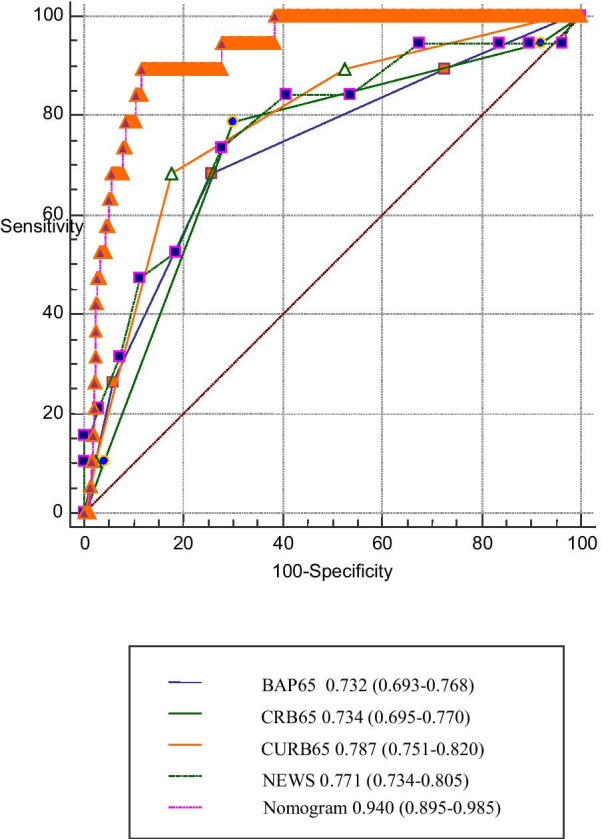
Receiver operating characteristic curves of the nomogram and other prognostic models. The AUC and the corresponding 95% confidence interval estimate are highlighted in black text

The AUCs for in-hospital mortality of BAP65, CRB65, CURB65, NEWS, and the nomogram were 0.732 (95% CI 0.693–0.768), 0.734 (95% CI 0.695–0.770), 0.787 (95% CI 0.751–0.820), 0.771 (95% CI 0.734–0.850) and 0.940 (95% CI 0.895–0.985) respectively (Fig. 5). For ICU admission and invasive mechanical ventilation, the AUCs of the nomogram were 0.7822 (95% CI 0.7408–0.8237) and 0.8044 (95% CI 0.7615–0.8473), respectively.

## Discussion

We developed and validated a nomogram for the individualized mortality prediction in patients with AECOPD with hypercapnic respiratory failure. This nomogram incorporated eight predictors: respiratory rate, PCO_2_, lactic acid, BUN, haemoglobin, PDW, platelet count, and concomitant pneumonia. All eight predictors could be readily accessed at the ED. The model allowed good discrimination with an AUC of 0.940 (95% CI 0.895–0.985) for in-hospital mortality. Additionally, the nomogram showed good performance for invasive mechanical ventilation in the ED and ICU admission. Moreover, it showed potential clinical utility. Compared with other prognostic models, including CRB65, CURB65, BAP65, and NEWS, the nomogram showed better performance in predicting the mortality risk of patients with AECOPD. In the subgroups of patients with different concomitant conditions, the model also showed a good predictive performance. To our knowledge, there has been no study that has developed a prediction model for assessing individual mortality risk in patients with AECOPD with hypercapnic respiratory failure.

Numerous factors are associated with AECOPD prognosis. However, there are often strong between-factor correlations, especially in laboratory tests, including PO_2_, oxygenation index, PDW, mean platelet volume, PCO_2_, PH, etc. Inconsistent results are yielded by incomplete collection of factors or use of different statistical methods. Based on other studies, we extensively collected factors readily available in ED that may be associated with patient outcomes. These included vital signs, state of consciousness, laboratory tests, and major comorbidities. Specifically, we considered factors that have recently attracted more attention, including D-dimer [20], NT-proBNP, troponin [21], red blood cell distribution width [22], platelet count [8], and mean platelet volume [10]. To construct the predictive model, 12 potential predictors were identified from 80 candidate variables by examining the predictor-outcome association through shrinking the regression coefficients using the LASSO method. In practice, it is often easier to implement models with fewer predictor variables. Most clinicians are unwilling to use large models requiring an extensive collection of information [23]. Therefore, we used the stepwise regression to reduce the number of variables to seven, which made the model more practical. The superior AUC suggested that this did not reduce the predictive performance of the model.

In addition to the number of predictors and the prediction accuracy, the variable availability should be considered in model establishment. Particularly in the ED, data access is limited by equipment, time, and patient conditions, including lung function, 6-min walk test, etc. There was no routine data collection on smoking, long-term oxygen treatment, and BMI, which are used for predicting patient 90-day or long-term survival, as well as re-exacerbation [11, 24–26]. In the ED, it is crucial to determine how to use the available data, quickly and effectively distinguish the criticality of the patients, and make the right treatment decision. Therefore, we mainly included variables readily available in ED; further, real-time data collection reflected the patient's current condition. Our nomogram involved 8 variables, which are all required for routine evaluation in the ED. Therefore, this model has a strong practical value. Furthermore, we made full use of the data to develop the model and used bootstrap for internal validation to prevent the loss of estimation precision in models developed on a data subset [24]. A nomogram can be used to predict individualized survival probability in patients with AECOPD [25], which provides a powerful tool for patients and health care professionals in making decisions.

The respiratory rate is an effective indicator for determining the lung condition and dyspnoea. An increased respiratory rate often indicates lung inflammation or gas exchange inefficiency; moreover, it predicts increased mortality, which is consistent with the study by Akhter [26, 27].

PCO_2_ has received increasing attention as an important prognostic factor for patients with AECOPD. Although there have been inconsistent reports [28–30], clinicians often consider hypercapnia as an indicator of more severe respiratory disease, worse prognosis, and higher complication risk from oxygen therapy including respiratory depression [7, 11, 13, 20, 28, 31–33]. Additionally, in clinical practice, patients with carbon dioxide retention have a higher mortality risk. Therefore, we enrolled patients with AECOPD with hypercapnia respiratory failure. In patients with oxygen-dependent COPD, PaCO_2_ (air) is an independent prognostic factor with a U-shaped association with mortality; specifically, the lowest mortality is observed at approximately 6.5 kPa with the mortality increasing at < 5.0 kPa and > 7.0 kPa [6]. In patients with AECOPD who had PCO_2_ ≥ 50 mmHg, there was a positive correlation of mortality risk with PCO_2_ levels, which is consistent with the study by Ahmadi [6].

Infection is among the main AECOPD causes. Among ED patients with suspected infection, there is an association of intermediate lactate elevation with moderate-to-high mortality risk [34]. Further, blood levels of lactic acid are affected by the effectiveness of non-invasive ventilation in the treatment of AECOPD with different severities [35]. In our study, 213 (35.44%) patients underwent non-invasive mechanical ventilation therapy. Therefore, the lactate level is another important prognostic factor. There is significant clinical utility in the dynamic monitoring of lactic acid for detecting changes in a patient’s condition [35].

BUN is another important marker of poor outcome in patients with AECOPD. Acute renal failure and elevated BUN levels at hospital admission were associated with higher hospital mortality [12].

The ability to transport oxygen is directly dependent on haemoglobin levels. Inadequate haemoglobin levels could aggravate tissue hypoxia and negatively affect the prognosis [36, 37]. Anaemia is a strong independent dyspnoea predictor in patients with COPD [36]. Haemoglobin levels are negatively associated with mortality in patients with AECOPD.

Platelet and platelet activity (including mean platelet volume, PDW, etc.) are associated with haemostasis and coagulation function [38–41]. Furthermore, there are associated with tumour development and inflammation [41–44]. Platelet count has a U-shaped association with increased risk of 3-year all-cause mortality in patients with COPD [8]. For analysis, we divided patients into those with platelet levels > 300 × 10^9^/L and < 100 × 10^9^/L. We found that patients with normal platelet levels had the lowest mortality risk while it was highest among patients with platelet levels < 100 × 10^9^/L. There have been inconsistent reports regarding the relationship between PDW and death. Şahin [45] reported no significant difference in PDW between patients with stable and acute exacerbation of COPD. Another study reported a protective role of PDW; further, inhaled corticosteroids/long-acting β2-agonists therapy increased PDW values in patients with COPD [46]. We found a negative correlation of PDW with mortality.

Respiratory viruses, airway bacteria are common causes of COPD exacerbations [47], patients with AECOPD often complicated with pneumonia. In the cohort we studied, 45.6% patients had pneumonia. Based on this clinical consideration, the variable of concomitant pneumonia was incorporated into the construction of the nomogram. The result shows that the nomogram can be applied to both AECOPD patients with pneumonia and without pneumonia.

This study has several limitations. First, this was a single-centre retrospective study; therefore, the results may not be generalizable to other settings and populations. Second, given the limitations in the ED, we did not collect other factors associated with AECOPD mortality, including lung function, BMI, smoking, and long-term oxygen treatment. Therefore, we could not determine the correlation between these factors and death. However, we built a model with good performance using parameters easily available in the ED. Moreover, the addition of other variables would not significantly improve the prediction effect of the model. Third, we only studied patients with hypercapnia respiratory failure. Further studies are required to determine whether the results apply to non-hypercapnia patients.

## Conclusion

In conclusion, this study presents a nomogram that incorporates respiratory rate, PCO_2_, lactic acid, BUN, haemoglobin, platelet distribution width, and platelet count.

It can be conveniently used by ED staff to grade patients with AECOPD faster, and accurately, and therefore manage patients more effectively in the complex environment of the ED, and enhancing the quality of patient care.

## Supplementary Information

**Additional file 1.** Other baseline demographic and clinical characteristics of the study participants.

## Data Availability

The datasets used in this study are available from the corresponding author upon reasonable request.

## Abbreviations

ED: Emergency departments
AUC: Area under the receiver operating curve
DCA: Decision curve analysis
APACHE-II: Acute physiology and chronic health evaluation-II score
LASSO: Least absolute shrinkage and selection operator
BUN: Blood urea nitrogen
