# Supplementary material for: Emergency admission parameters for predicting in-hospital mortality in patients with acute exacerbations of chronic obstructive pulmonary disease with hypercapnic respiratory failure
Source: BMC Pulm Med. 2021 Aug 6;21:258. doi: 10.1186/s12890-021-01624-1 (PMC8349105; doi:10.1186/s12890-021-01624-1)
Supplement: Supplementary file 1 — Additional file 1. Other baseline demographic and clinical characteristics of the study participants. [file 12890_2021_1624_MOESM1_ESM.doc]

**Additional file 1: Table S1:** Other baseline demographic and clinical characteristics of the study participants.

| Variable | Death during hospitalization | | P |
| --- | --- | --- | --- |
|  | Yes (n=19) | No (n=582) |  |
| **Baseline characteristics** |  |  |  |
| Heart rate(beat/min) | 110.5 ± 32.4 | 106.2 ± 20.8 | 0.385 |
| systolic blood pressure (mmHg) | 138.2 ± 42.0 | 141.5 ± 28.5 | 0.616 |
| diastolic blood pressure (mmHg) | 75.7 ± 24.6 | 78.4 ± 17.1 | 0.498 |
| **Laboratory findings** |  |  |  |
| Arterial oxygen saturation (%) | 84.6 ± 12.9 | 81.6 ± 17.5 | 0.460 |
| Total carbon dioxide (mmol/L) | 30.0 ± 10.8 | 31.4 ± 6.4 | 0.378 |
| Actual Bicarbonate Radical (mmol/L) | 31.2 ± 11.5 | 34.3 ± 6.7 | 0.060 |
| Carbonyl haemoglobin (%) | 1.4 ± 0.5 | 1.6 ± 0.7 | 0.112 |
| Blood oxygen content (mmol/L) | 6.1 ± 1.3 | 6.7 ± 1.8 | 0.148 |
| Oxyhaemoglobin (%) | 83.0 ± 12.7 | 79.8 ± 17.1 | 0.423 |
| Troponin I, ng/mL | 0.0 (0.0-0.1) | 0.0 (0.0-0.1) | 0.799 |
| Glutamic oxalacetic transaminase (IU/L) | 39.2 (29.9-65.5) | 29.0 (22.0-43.3) | 0.485 |
| Creatine phosphokinasem (IU/L) | 82.3 (42.2-135.4) | 61.0 (40.1-97.4) | 0.106 |
| MB isoenzymes of creatine kinase (IU/L) | 26.6 (13.9-30.3) | 18.6 (12.6-26.9) | 0.211 |
| Alanine aminotransferase (IU/L) | 20.9 (15.8-30.5) | 19.1 (11.5-31.2) | 0.446 |
| Sodium (mmol/L) | 137.3 ± 4.7 | 139.1 ± 5.5 | 0.176 |
| Chlorine (mmol/L) | 97.7 ± 6.0 | 99.4 ± 6.2 | 0.265 |
| Calcium (mmol/L) | 2.2 ± 0.2 | 2.2 ± 0.2 | 0.336 |
| Glucose (mmol/L) | 11.6 ± 6.0 | 9.1 ± 3.5 | 0.106 |
| Total protein (g/L) | 69.5 ± 5.5 | 71.0 ± 7.4 | 0.394 |
| Total bilirubin (μmol/L) | 10.0 (8.8-12.8) | 12.8 (9.2-18.9) | 0.618 |
| Direct bilirubin (μmol/L) | 2.9 (0.0-5.9) | 2.4 (0.0-4.3) | 0.774 |
| [Monocyte](#keyfrom=E2Ctranslation) (%) | 5.8 (3.6-7.6) | 6.3 (4.5-8.4) | 0.265 |
| [Monocyte](#keyfrom=E2Ctranslation) count (*10^9^/L) | 0.7 (0.4-1.1) | 0.6 (0.4-0.8) | 0.246 |
| L[lymphocyte](#keyfrom=E2Ctranslation) (%) | 7.8 (6.2-15.8) | 10.8 (5.5-16.8) | 0.570 |
| Basophilic granulocyte (%) | 0.1 (0.1-0.3) | 0.2 (0.0-0.3) | 0.691 |
| Basophilic granulocyte count (*10^9^/L) | 0.0 (0.0-0.0) | 0.0 (0.0-0.0) | 0.168 |
| Eosinophils (%) | 0.2 (0.0-0.6) | 0.3 (0.0-1.3) | 0.389 |
| Eosinophils count (*10^9^/L) | 0.0 (0.0-0.2) | 0.0 (0.0-0.1) | 0.715 |
| Neutrophils (%) | 81.2 ± 10.9 | 78.3 ± 13.6 | 0.365 |
| Erythrocyte distribution width (%) | 14.4 ± 2.3 | 14.3 ± 2.1 | 0.904 |
| Thrombocytocrit (%) | 0.2 ± 0.1 | 0.2 ± 0.1 | 0.386 |
| Activated partial thromboplastin time (s) | 34.1 ± 6.9 | 33.0 ± 6.2 | 0.454 |
| Thrombin time (s) | 14.8 ± 1.9 | 14.3 ± 2.0 | 0.222 |
| International Normalized Ratio | 1.3 ± 0.3 | 1.1 ± 0.2 | 0.005 |
| Prothrombin time (s) | 14.4 ± 3.6 | 12.6 ± 2.8 | 0.005 |
| D-dimer (μg/L) **, n (%)** |  |  | 0.050 |
| <500 | 1 (5.3) | 135 (24.8) |  |
| >=500 | 18 (94.7) | 409 (75.2) |  |
| Procalcitonin (ng/mL) **, n (%)** |  |  | 0.154 |
| <=2 | 10 (83.3) | 300 (93.8) |  |
| >2 | 2 (16.7) | 20 (6.2) |  |
| NT-pro-BNP ( pg/mL) **, n (%)** |  |  | 0.258 |
| <450 | 3 (16.7) | 159 (28.9) |  |
| >=450 | 15 (83.3) | 391 (71.1) |  |
| **Comorbidities, n (%)** |  |  |  |
| Stroke | 0 (0.0) | 8 (1.4) | 0.607 |
| Haematological system diseases | 3 (0.5) | 0 (0.0) | 0.754 |
| Upper gastrointestinal bleeding | 13 (2.2) | 0 (0.0) | 0.510 |
| Neuromuscular disease | 3 (0.5) | 0 (0.0) | 1.000 |
| Chronic liver disease | 10 (1.7) | 0 (0.0) | 0.564 |

**Additional file 1: Table S2:** The receiver operating characteristic curves and internal validation of the nomogram in subgroups splited by the time patient admitted to ED.

| Subgroups | Original AUC | 95% CI | Sensitivity(%) | Specificity(%) | PPV (%) | NPV (%) | Corrected AUC ^a^ | 95% CI |
| --- | --- | --- | --- | --- | --- | --- | --- | --- |
| January 2018 to May 2019 | 0.930 | 0.879- 0.981 | 0.886 | 0.900 | 0.225 | 0.996 | 0.924 | 0.843- 0.968 |
| June 2019 to October 2020 | 0.919 | 0.837- 1.000 | 0.889 | 0.888 | 0.200 | 0.996 | 0.908 | 0.778- 0.978 |

CI, confidence interval; PPV, positive predictive value; NPV, negative predictive value.

^a^ Using bootstrap 500.

**Additional file 1: Table S3:** The receiver operating characteristic curves and internal validation of the nomogram in all AECOPD patients presenting to ED.

|  | Original AUC | 95%CI | Sensitivity(%) | Specificity(%) | PPV (%) | NPV (%) | CorrectedAUC ^a^ | 95%CI |
| --- | --- | --- | --- | --- | --- | --- | --- | --- |
| Nomogram | 0.895 | 0.836- 0.955 | 0.885 | 0.843 | 0.154 | 0.996 | 0.889 | 0.818- 0.942 |

CI, confidence interval; PPV, positive predictive value; NPV, negative predictive value.

^a^ Using bootstrap 500.

**Additional file 1: Table S4:** The receiver operating characteristic curves and internal validation of the nomogram in subgroups with or without concomitant diseases.

| Concomitant | Original AUC | 95%CI | Sensitivity(%) | Specificity(%) | PPV (%) | NPV (%) | Corrected AUC ^a^ | 95%CI |
| --- | --- | --- | --- | --- | --- | --- | --- | --- |
| Pneumonia |  |  |  |  |  |  | 0.889 |  |
| No | 0.932 | 0.877- 0.987 | 0.917 | 0.893 | 0.256 | 0.996 | 0.924 | 0.845- 0.974 |
| Yes | 0.914 | 0.835- 0.992 | 0.857 | 0.888 | 0.171 | 0.996 | 0.905 | 0.756- 0.969 |
| Heart failure or coronary heart disease |  |  |  |  |  |  |  |  |
| No | 0.934 | 0.885- 0.983 | 0.875 | 0.896 | 0.241 | 0.995 | 0.926 | 0.855- 0.974 |
| Yes | 0.891 | 0.828- 0.955 | 1.000 | 0.867 | 0.143 | 1.000 | 0.885 | 0.830- 0.935 |
| Hydropneumothorax |  |  |  |  |  |  |  |  |
| No | 0.924 | 0.879- 0.969 | 0.895 | 0.883 | 0.207 | 0.996 | 0.917 | 0.861- 0.959 |

CI, confidence interval; PPV, positive predictive value; NPV, negative predictive value.

^a^ Using bootstrap 500.
